# Performance Characteristics of Putative Tests for Subclinical Chorioamnionitis

**DOI:** 10.1155/S1064744901000345

**Published:** 2001

**Authors:** Rodney K. Edwards, Penny Clark, Gregory J. Locksmith, Patrick Duff

**Affiliations:** 1 Department of Obstetrics and Gynecology University of Florida College of Medicine PO Box 100294 1600 SW Archer Road Gainesville FL 32610-0294 USA; 2 Department of Obstetrics, Gynecology and Reproductive Sciences University of Texas Medical Branch Galveston TX USA

## Abstract

*Objective:* To evaluate amniotic fluid glucose, matrix metalloproteinase (MMP)-9, interleukin (IL)-6, and IL-12 for diagnosing subclinical chorioamnionitis in women with preterm labor.

*Methods:* Forty-four women in preterm labor at  22–35  weeks gestation with suspected subclinical chorioamnionitis underwentamniocentesis.Amniotic fluid analysis included Gram stain, culture, and determination of glucose, MMP-9, IL-6, and IL-12 concentrations. Median values of these analytes were compared using the Mann-Whitney U test. Sensitivity, specificity, and positive and negative predictive values were calculated for tests using a positive amniotic fluid culture or delivery within 24 hours as the key outcome variables

*Results:* Amniotic fluid concentrations of glucose, MMP-9, and IL-6 correlated closely with positive culture or delivery within 24 hours. IL-12 concentrations did not correlate with either a positive culture or delivery within 24 hours.

*Conclusions:* Amniotic fluid glucose, MMP-9, and IL-6 reliably predict microbial invasion of the amniotic cavity or imminent delivery. IL-12 values did not correlate with amniotic fluid culture results or imminent delivery.


*Infect Dis Obstet Gynecol 2001;9:209–214*

### Performance characteristics of putative tests for subclinical chorioamnionitis

**Rodney K. Edwards[1], Penny Clark[1], Gregory J. Locksmith[2] and Patrick Duff[1]**

[1]*Department of Obstetrics and Gynecology, University of Florida College of Medicine, Gainesville, FL*
[2]*Department of Obstetrics, Gynecology and Reproductive Sciences, University of Texas Medical Branch, Galveston, TX*

***Objective:*** To evaluate amniotic fluid glucose, matrix metalloproteinase (MMP)-9, interleukin (IL)-6, and IL-12 for diagnosing subclinical chorioamnionitis in women with preterm labor.
***Methods:*** Forty-four women in preterm labor at 22–35 weeks gestation with suspected subclinical chorioamnionitis underwent amniocentesis. Amniotic fluid analysis included Gram stain, culture, and determination of glucose, MMP-9, IL-6, and IL-12 concentrations. Median values of these analytes were compared using the Mann-Whitney U test. Sensitivity, specificity, and positive and negative predictive values were calculated for tests using a positive amniotic fluid culture or delivery within 24 hours as the key outcome variables.
***Results:*** Amniotic fluid concentrations of glucose, MMP-9, and IL-6 correlated closely with positive culture or delivery within 24 hours. IL-12 concentrations did not correlate with either a positive culture or delivery within 24 hours.
***Conclusions:*** Amniotic fluid glucose, MMP-9, and IL-6 reliably predict microbial invasion of the amniotic cavity or imminent delivery. IL-12 values did not correlate with amniotic fluid culture results or imminent delivery.

Key words: Cytokines, Preterm Labor, Interleukin-6, Interleukin-12, Matrix Metalloproteinase-9

Intrauterine infection is an important cause of spontaneous preterm birth[1,2]. Chorioamnionitis is generally diagnosed based on clinical criteria; however, even subclinical chorioamnionitis can result in preterm labor. Microbial invasion of the amniotic cavity increases maternal and neonatal complications. For instance, women with subclinical chorioamnionitis are more likely to develop clinical chorioamnionitis, rupture of membranes, and failure of tocolysis[3-6]. Neonates born to mothers with subclinical chorioamnionitis have increased rates of infection and other morbidities[5,7]. Therefore, accurate identification of the woman in preterm labor with subclinical chorioamnionitis is important.

Microbiologic culture of amniotic fluid (AF) obtained by transabdominal amniocentesis has been the gold standard for diagnosing microbial invasion of the amniotic cavity. The primary disadvantages of this technique are delay in reporting of test results and low sensitivity. From the time amniocentesis has been performed, 2 to 3 days may elapse before a positive or negative culture result is obtained. Sensitivity is diminished

Correspondence to: Rodney K. Edwards, MD, Department of Obstetrics and Gynecology, University of Florida College of Medicine, PO Box 100294, 1600 SW Archer Road, Gainesville, FL 32610-0294, USA

by suboptimal culture techniques and by exacting laboratory requirements needed to culture certain organisms, such as anaerobes or mycoplasma species. Additionally, infection of the decidua or membranes may exist in the absence of cultivable organisms in the AF. Hitti and colleagues[8] showed that a broad-spectrum bacterial 16S rRNA polymerase chain reaction (PCR) assay was more sensitive than culture for detecting organisms in AF. Although PCR results can be obtained more quickly than culture, this technology is not readily available for clinical use and is subject to contamination. Although PCR is more sensitive than culture, this technique also fails to detect organisms located solely in the fetal membranes or decidua.

Other tests that have been used to diagnose intrauterine infection include AF white blood cell count, Gram stain, glucose concentration, and interleukin (IL)-6 concentration. The most accurate predictor of positive AF culture among these tests is an elevated IL-6 concentration[9]. However, this test has a low positive predictive value (perhaps due to the limited sensitivity of culture), is relatively expensive, and is not readily available in most hospitals. Amniotic fluid glucose analysis may be slightly less sensitive than IL-6, but it is less expensive, can be obtained rapidly, and is widely available.

A less well-established marker of subclinical chorioamnionitis is matrix metalloproteinase (MMP)-9. This substance is one of the gelatinases, enzymes involved in the degradation of collagen, basement membrane components, and proteoglycans[10,11]. In an investigation utilizing the same cohort that we report on here, MMP-9 proved to reliably predict positive AF culture or imminent delivery[12]. However, like IL-6, MMP-9 is relatively expensive and is not readily available for clinical use.

IL-12 is a cytokine that, to our knowledge, has not been evaluated in AF as a potential marker of subclinical chorioamnionitis. IL-12 is produced by several cell types, including macrophages, in response to infectious agents and is a central mediator of the cell-mediated immune response[13–15].

We are unaware of any studies comparing the predictive value of all of these markers for subclinical chorioamnionitis. Therefore, the objective of our investigation was to compare the sensitivity, specificity, positive predictive value (PPV), and negative predictive value (NPV) of AF glucose, MMP-9, IL-6, and IL-12 for diagnosing bacterial infection of the AF in women with preterm labor, intact membranes, and signs suggestive of subclinical chorioamnionitis.

### SUBJECTS AND METHODS

Women who presented to the Labor and Delivery Unit at Shands Hospital at the University of Florida at 22–35 weeks gestation with preterm labor and the clinical suspicion of subclinical chorioamnionitis underwent amniocentesis. The suspicion of subclinical chorioamnionitis was based on the presence of one of the following: low-grade maternal temperature elevation (less than 38°C), isolated fetal tachycardia, or poor response to tocolysis. Women were approached for inclusion in this prospective cohort study if they had documented cervical change, at least 4 contractions per hour, and a cervix that was at least 2 centimeters dilated or 80% effaced. If these women had ruptured membranes, cervical dilation of more than 5 centimeters, or had taken antibiotics within the preceding 7 days, they were excluded from the study. The Health Center Institutional Review Board approved the investigation. Between July 1, 1996 and May 31, 1998, 44 women met the inclusion criteria and consented in writing to participate in the study.

The procedure for handling AF samples has been described in another study[12]. Briefly, after collection by transabdominal amniocentesis, AF was analyzed by Gram stain, culture for aerobes, anaerobes, and mycoplasmas, and glucose concentration. The results of these tests were used to make decisions regarding clinical treatment of patients. An additional aliquot of AF was centrifuged, and the supernatant stored at −80°C.

The stored AF samples were analyzed for MMP-9 concentration using gelatin zymography and a commercially available enzyme-linked immunosorbent assay (ELISA) system[12]. We used quantitative sandwich enzyme immunoassays (R&D Systems, Minneapolis, MN) to measure total IL-6 and IL-12 concentrations in the same stored AF samples. Concentrations of IL-6 and

IL-12 were determined by interpolation from a curve constructed from known standards. Samples were analyzed undiluted. When concentrations were greater than the highest standard concentration, samples were diluted 1 : 100 and 1 : 1000 for final analysis. All samples were analyzed in duplicate.

All women received intravenous magnesium sulfate as first-line tocolysis. If AF glucose, Gram stain, or culture were positive, tocolysis was discontinued. None of the other AF test results were available for use in clinical decision making. Finally, antimicrobial therapy was initiated only for prophylaxis against early-onset neonatal sepsis with group B streptococcus or treatment of clinical chorioamnionitis.

For the analysis of demographic variables, we used the Yates-corrected z test. The Mann-Whitney U test was used to compare median values of MMP-9, IL-6, and IL-12 between subjects with and without positive AF cultures. Sensitivity, specificity, PPV, and NPV were calculated for AF glucose concentration, Gram stain, MMP-9, IL-6, and IL-12 using a positive AF culture as the principal outcome variable. Ninety-five percent confidence intervals (CI) were calculated for each of these values. To account for cases of probable subclinical chorioamnionitis with negative cultures, we recalculated the performance characteristics of each marker changing the key outcome variable to positive culture, positive Gram stain, or spontaneous preterm birth within 24 hours of amniocentesis. Neither the interval from amniocentesis to hospital discharge nor the duration of tocolysis were specifically evaluated.

### RESULTS

The demographic characteristics of this cohort have been reported[12]. In brief, of the 44 women in the study, six [14% (95% CI 4, 24)] had positive AF cultures. Compared to those with negative AF cultures, women with positive AF cultures were more likely to be less than 28 weeks gestational age and to be African-American. There were no significant differences between these two groups in terms of age, parity, or cervical dilation at presentation.

The median MMP-9 concentration in the AF of women with positive AF cultures was 557 ng/ml (interquartile range 352–730 ng/ml), as compared to 0 ng/ml (interquartile range 0–0 ng/ml) for those with negative cultures ($p = 0.003$)[12]. Similarly, median IL-6 concentrations in AF were significantly higher in women with positive AF cultures (53.8 ng/ml, interquartile range 8.9–129.7) than in those with negative AF cultures (0.77 ng/ml, interquartile range 0.26–2.29) ($p = 0.02$). However, median IL-12 concentrations in AF were similar in women with positive (70.6 pg/ml, interquartile range 64.5–74.1) and negative (64.5 pg/ml, interquartile range 50.4–68.0) AF cultures ($p = 0.66$).

Using positive AF culture as the criterion for the diagnosis of subclinical chorioamnionitis, performance statistics for IL-6, MMP-9, and glucose are shown in Table 1. The results are dichotomized using the lower limit of the interquartile range for IL-6 and MMP-9 as cut-off values. For AF glucose, we used a threshold (15 mg/dl) that has been established by others and is in clinical use at our center. Values below this threshold were considered positive.

Details of subjects with positive cultures, glucose, IL-6, or MMP-9 are shown in Table 2. Premature rupture of membranes, clinical chorioamnionitis, or delivery within 24 hours of amniocentesis occurred in every case with a positive culture except one. This woman's AF sample grew *Xanthomonas maltophilia*, a ubiquitous organism

**Table 1** Performance statistics (95% confidence intervals) for AF concentrations of IL-6, MMP-9, and glucose using positive AF culture as the key outcome variable

| *Test* | *Sensitivity* | *Specificity* | *PPV* | *NPV* |
|---|---|---|---|---|
| IL-6 (> 8.9 ng/ml) | 83% (53, 100) | 89% (80, 99) | 56% (23, 88) | 97% (92, 100) |
| MMP-9 (> 351 ng/ml) | 83% (53, 100) | 92% (84, 100) | 63% (29, 96) | 97% (92, 100) |
| Glucose (< 15 mg/dl) | 83% (53, 100) | 87% (75, 99) | 56% (23, 88) | 97% (92, 100) |

AF, amniotic fluid; PPV, positive predictive value; NPV, negative predictive value

**Table 2** Characteristics of women with positive AF cultures, glucose, IL-6, or MMP-9

| *GA* (weeks) | *Culture* | *Glucose* (mg/dl) | *IL-6* (ng/ml) | *MMP-9* (ng/ml) | *Pregnancy outcome* |
|---|---|---|---|---|---|
| 25 | *Lactobacillus* | 13 | 9.0 | 352 | PROM and Cesarean delivery within 48 h |
| 22 | *Fusobacterium* | < 10 | 26.9 | 730 | Treated with antibiotics, PTL and SVD 11 days after amniocentesis |
| 33 | *Xanthomonas maltophilia* | 38 | 0.1 | 0 | No treatment, SVD at 37 weeks |
| 24 | *Streptococcus viridans* | < 10 | 80.7 | 452 | PTL and SVD in < 24 h |
| 22 | *Ureaplasma urealyticum* | < 10 | 423.6 | 662 | Labor induced due to clinical chorioamnionitis, SVD in < 24 h |
| 31 | *Mycoplasma hominis* | < 10 | 129.7 | 1066 | PTL and SVD in < 24 h |
| 34 | Negative | 12 | 36.5 | 340 | PTL and SVD in < 24 h |
| 34 | Negative | < 10 | 66.8 | 933 | PTL and SVD in < 24 h |
| 34 | Negative | 14 | 1.6 | 0 | Labor induced after PROM at 36 weeks, SVD 15 days after amniocentesis |
| 26 | Negative | < 10 | 233.4 | 860 | Labor induced due to GNR on AF Gram stain; SVD in < 24 h |
| 29 | Negative | 23 | 86.4 | 0 | PTL and SVD in < 24 h |

AF, amniotic fluid; GA, gestational age; PROM, premature rupture of membranes; PTL, spontaneous preterm labor; SVD, spontaneous vaginal delivery

**Table 3** Performance statistics (95% confidence intervals) for AF concentrations of IL-6, MMP-9, and glucose using positive AF culture, positive AF Gram stain, or spontaneous preterm birth within 24 hours of amniocentesis as the key outcome variables.

| *Test* | *Sensitivity* | *Specificity* | *PPV* | *NPV* |
|---|---|---|---|---|
| IL-6 (> 8.9 ng/ml) | 100% (94, 100) | 100% (97, 100) | 100% (94, 100) | 100% (97, 100) |
| MMP-9 (> 351 ng/ml) | 78% (51, 100) | 100% (97, 100) | 100% (93, 100) | 94% (86, 100) |
| Glucose (< 15 mg/dl) | 90% (71, 100) | 87% (75, 99) | 89% (69, 100) | 97% (93, 100) |

AF, Amniotic fluid; PPV, positive predictive value; NPV, negative predictive value found on skin, in hospital water conduits, and in deionized water[16]. This woman delivered at term, four weeks after amniocentesis, without any treatment. We judged this organism to be a contaminant. Considering this case as having a negative culture result and expanding the criteria for diagnosis of probable subclinical chorioamnionitis to include positive AF culture, positive AF Gram stain, or spontaneous preterm birth within 24 hours of amniocentesis, we recalculated the performance statistics for IL-6, MMP-9, and glucose. These results are shown in Table 3.

### DISCUSSION

The results of our study are in keeping with the results of many others in that elevated AF IL-6 concentrations correlated with positive AF culture or imminent delivery[17–19]. The performance statistics for IL-6 were not significantly better than for AF glucose or MMP-9, however our sample size was too small to detect clinically important differences between methods. Although IL-12 is produced by macrophages and other cell types in response to microorganisms and is thought to be key in bridging the innate and cellular immune response[13–15], we found no relationship between AF IL-12 concentration and either positive AF culture or imminent delivery.

We observed that AF glucose, IL-6, and MMP-9 correlated with one another and with outcome. All three of these tests were positive in seven cases. Each of these cases had either positive culture results or delivered within 24 hours of amniocentesis. Another woman with a negative AF culture result who delivered within 24 hours had a positive glucose and IL-6 and an MMP-9 concentration just below the threshold to be considered positive.

Although inflammatory markers can be positive in the absence of infection or the presence of a remote site of infection, the most likely explanation for cases with positive inflammatory markers and negative AF cultures is the inability of AF culture to detect all cases of intrauterine infection. Possible explanations for negative AF cultures when bacteria are present in the uterus include: (1) the antibacterial properties of AF, (2) the low sensitivity of culture, (3) the presence of unculturable or difficult to culture organisms, and (4) cases in which bacteria are located within the membranes or between the membranes and decidua. Clearly, by itself, AF culture is an inadequate standard for detecting intrauterine infection in the setting of preterm labor. Future investigations that utilize PCR to identify organisms in AF may reduce the discrepancy between inflammatory markers and presence of organisms in AF.

Because of issues related to cost and availability, tests for IL-6 and MMP-9 concentrations in AF are not readily available for clinical use. Our data indicate that AF glucose determination is a reasonable surrogate for these markers. Further investigation is needed to justify making other markers clinically available. Specifically, defining the temporal and mechanistic roles of the various inflammatory mediators in the pathophysiology of preterm labor is needed. However, the optimal clinical management of a patient in preterm labor at a very early gestational age with subclinical chorioamnionitis has yet to be determined. If it could be shown that tests for certain inflammatory markers become positive before microorganisms can be detected in AF and that the presence of these markers is reliably linked to preterm delivery and/or neonatal morbidity, these tests would have practical application to clinical practice. Finally, studies are needed to answer the more fundamental question of why some patients, but not others, develop intrauterine infections that lead to preterm labor.

### REFERENCES

1. Gibbs RS, Romero R, Hillier SL, *et al.* A review of premature birth and subclinical infection. *Am J Obstet Gynecol* 1992;166:1515–28
2. Gomez R, Romero R, Edwin SS, David C. Pathogenesis of preterm labor and preterm premature rupture of membranes associated with intra-amniotic infection. *Infect Dis Clin North Am* 1997;11:135–76
3. Romero R, Mazor M. Infection and preterm labor. *Clin Obstet Gynecol* 1988;31:553–84
4. Hameed C, Tejani N, Verma UL, Archbald F. Silent chorioamnionitis as a cause of preterm labor refractory to tocolytic therapy. *Am J Obstet Gynecol* 1984;149:726–30
5. Duff P, Kopelman JN. Subclinical intra-amniotic infection in asymptomatic patients with refractory preterm labor. *Obstet Gynecol* 1989;69:756–9
6. Dunlow SG, Duff P. Microbiology of the lower genital tract and amniotic fluid in asymptomatic preterm patients with intact membranes and moderate to advanced degrees of cervical effacement and dilation. *Am J Perinatol* 1990;7:235–8
7. Watts DH, Krohn MA, Hillier SL, Eschenbach DA. The association of occult amniotic fluid infection with gestational age and neonatal outcome among women in preterm labor. *Obstet Gynecol* 1992;79:351–7
8. Hitti J, Riley DE, Krohn MA, *et al.* Broad-spectrum bacterial rDNA polymerase chain reaction assay for detecting amniotic fluid infection among women in premature labor. *Clin Infect Dis* 1997;24:1228–32
9. Romero R, Yoon BH, Mazor M, *et al.* The diagnostic and prognostic value of amniotic fluid white blood cell count, glucose, interleukin-6, and Gram stain in patients with preterm labor and intact membranes. *Am J Obstet Gynecol* 1993;169:805–16
10. Matrisian L. Matrix metalloproteinases and their inhibitors in matrix remodeling. *Trends Genet* 1990;6:121–5
11. Woessner JF. Matrix metalloproteinases and their inhibitors in connective tissue remodeling. *FASEB J* 1991;5:2145–54
12. Locksmith GJ, Clark P, Duff P, Schultz GS. Amniotic fluid matrix metalloproteinase-9 levels in women with preterm labor and suspected intra-amniotic infection. *Obstet Gynecol* 1999;94:l–6

13. Trinchieri G. Interleukin-12 and its role in the generation of $T_H1$ cells. *Immunol Today* 1993;14: 335–8
14. Scott P. IL-12: initiation cytokine for cell-mediated immunity. *Science* 1993;260:496–7
15. Locksley RM. Interleukin-12 in host defense against microbial pathogens. *Proc Natl Acad Sci USA* 1993;90:5879–80
16. Gilardi GL. Pseudomonas. In Lenette EH, ed. *Manual of Clinical Microbiology*. 4th edn. Washington DC: *Am Soc Clin Microbiol*, 1985: 350–72
17. Hillier SL, Witkin SS, Krohn MA, *et al.* The relationship of amniotic fluid cytokines and preterm delivery, amniotic fluid infection, histologic chorioamnionitis, and chorioamnion infection. *Obstet Gynecol* 1993;81:941–8
18. Saito S, Kasahara T, Kato Y, *et al.* Elevation of amniotic fluid interleukin (IL)-6, IL-8, and granulocyte colony stimulating factor (G-CSF) in term and preterm parturition. *Cytokine* 1993;5: 81–8
19. Yoon BH, Romero R, Park JS, *et al.* Microbial invasion of the amniotic cavity with *Ureaplasma urealyticum* is associated with a robust host response in fetal, amniotic, and maternal compartments. *Am J Obstet Gynecol* 1998;179:1254–60

RECEIVED 05/10/01; ACCEPTED 08/24/01
